# Heavy Alcohol Consumption with Alcoholic Liver Disease Accelerates Sarcopenia in Elderly Korean Males: The Korean National Health and Nutrition Examination Survey 2008-2010

**DOI:** 10.1371/journal.pone.0163222

**Published:** 2016-09-21

**Authors:** Do Seon Song, U Im Chang, Sooa Choi, Yun Duk Jung, Kyungdo Han, Seung-Hyun Ko, Yu-Bae Ahn, Jin Mo Yang

**Affiliations:** 1 Division of Hepatology, Department of Internal Medicine, St. Vincent’s Hospital, College of Medicine, The Catholic University of Korea, Suwon, Korea; 2 Department of Biostatics, College of Medicine, The Catholic University of Korea, Seoul, Korea; 3 Division of Endocrinology and Metabolism, Department of Internal Medicine, St. Vincent’s Hospital, College of Medicine, The Catholic University of Korea, Suwon, Korea; Ehime University Graduate School of Medicine, JAPAN

## Abstract

**Background and Aim:**

Although a few studies have reported that sarcopenia is associated with alcoholic liver disease (ALD), no studies have investigated this association in a large sample representative of the elderly Korean population.

**Methods:**

This was a cross-sectional study that used data from the Fourth and Fifth Korean National Health and Nutrition Examination Surveys (KNHANES) on subjects aged 65 years and older. Sarcopenia was defined as a skeletal muscle index (SMI) more than 1 SD below the gender-specific mean for young adults; SMI was calculated as the appendicular muscle mass divided by height squared (ASM/Ht^2^). Heavy alcohol consumption was defined as consuming at least 210 g/week, and elevated liver enzymes were defined as alanine aminotransferase levels of at least 32 U/L or aspartate aminotransferase levels of at least 34 U/L. ALD was defined as heavy alcohol consumption and elevated liver enzymes.

**Results:**

The mean age of the 1,151 elderly males was 71.6 ± 0.2 years, and the prevalence of heavy alcohol consumption was 11.8% (136 subjects). SMI did not differ between the non-heavy and heavy alcohol consumer groups (7.1 ± 0.0 kg/m^2^ vs. 7.3 ± 0.1 kg/m^2^, respectively, *P* = 0.145). However, after stratifying by the presence of liver disease and heavy alcohol consumption and adjusting for other confounders in the multivariate logistic regression, SMI was significantly lower among heavy alcohol consumers with ALD (all *P* < 0.05). Additionally, two-way ANOVA showed a significant interaction between heavy alcohol consumption and liver disease (*P* = 0.011).

**Conclusion:**

Sarcopenia was accelerated in the elderly male ALD group, with a significant interaction between alcohol consumption and liver disease.

## Introduction

Alcohol consumption is a leading cause of global morbidity and mortality and is estimated to cause 2.7 million deaths worldwide each year[1]. Alcoholism is associated with more than 60 medical conditions as well as with accidental injuries[2]. The burden of alcoholic liver disease (ALD) is large, representing 0.9% of all global deaths and 47.9% of all liver cirrhosis deaths[3]. ALD is the third most common etiology of new waitlist registrants for liver transplant in the United States [4] and the most common cause of acute-on-chronic liver failure in Korea[5]. Chronic hepatitis C remains the leading cause of cirrhosis and hepatocellular carcinoma in Western areas. However, as potent antiviral agents are now available, the burden of viral hepatitis is expected to decrease[6], leading to an increased importance of ALD.

Malnutrition is a major complication of ALD and has been studied in patients with alcoholic hepatitis in particular. Malnutrition is associated with increased morbidity and mortality, although adequate nutritional support is known to improve nutritional status, reduce complications, and prolong survival in ALD[7, 8]. Therefore, assessing nutritional status is essential in patients with ALD. However, it is difficult to obtain accurate assessments of nutritional status in ALD. The fluid retention frequently observed in these patients confounds changes in body weight, and laboratory variables, such as albumin, prealbumin, and transferrin, lack accuracy because they are synthesized in the liver[9]. Therefore, the assessment of sarcopenia is emerging as a novel, accurate, objective marker of nutritional status in patients with liver disease[10, 11].

Sarcopenia is a syndrome characterized by a progressive and generalized loss of skeletal muscle mass and strength[12]. Although sarcopenia is associated with aging, it can also occur as a result of chronic disease and malignancy[13]. Sarcopenia is a common complication of liver disease and is associated with decreased functional capacity and a higher risk of morbidity and mortality[11, 14]. Alcohol consumption is also associated with skeletal muscle wasting and alcoholic myopathy through a complex series of mechanisms[15–17]. Furthermore, severe muscle loss contributes to worse outcomes in alcoholic hepatitis and liver cirrhosis[18]. Although a loss of muscle mass has been identified in individuals with alcohol use disorders, very little population research has been performed on the impact of alcohol consumption on sarcopenia. Kim et al. found no difference in the proportion of alcohol consumption between subjects with and without sarcopenia[19]. However, the distribution of high alcohol consumption was relatively low in the Korean population they studied, with the highest category of alcohol consumption being > 7 drinks for > 2 days/week, and they did not consider the presence of liver disease.

Therefore, we conducted this study to investigate the association between sarcopenia and ALD in a large sample representative of the Korean population.

## Methods

### Study population

**Fig 1:**
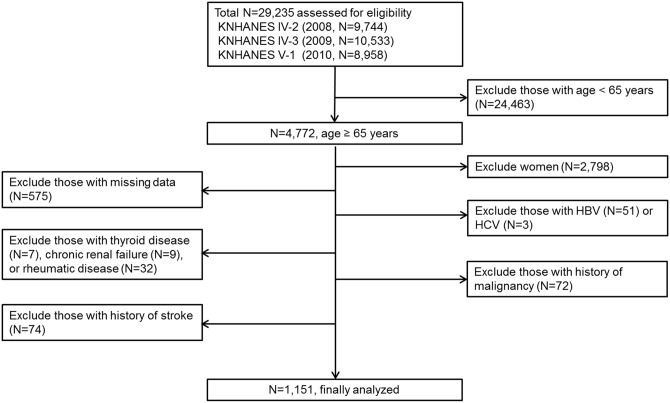
Flow diagram of subject inclusion and exclusion. Of the total subjects (N = 29,235), 1,151 elderly male participants were included. Abbreviations: HBV, hepatitis B virus; HCV, hepatitis C virus.

The Korean National Health and Nutrition Examination Survey (KNHANES) is a cross-sectional nationally representative health and nutrition examination survey conducted by the Korean Centers for Disease Control and Prevention. These surveys have been conducted periodically since 1998 to assess the health and nutritional status of the non-institutionalized civilian population in Korea. Additional details regarding the study design and methods are provided elsewhere[20]. This study used data from the Fourth KNHANES (KNHANES IV), collected from 2008 through 2009, and from the Fifth KNHANES (KNHANES V), collected during 2010. A total of 29,235 individuals participated in the surveys; of those, 24,463 individuals under the age 65 and 2,798 females were excluded due to the small number of heavy drinkers. To form an alcoholic liver disease group that was not influenced by other causes, we excluded 823 participants using the following exclusion criteria: missing data (n = 575); positive hepatitis B surface antigen tests (n = 37); physician-diagnosed chronic liver disease, including chronic hepatitis B (n = 14) or C (n = 3), chronic renal failure (n = 9), rheumatic disease (n = 32), and thyroid disease (n = 7); and history of malignancy (n = 72) or stroke (n = 74). Finally, a total of 1,151 elderly Korean male participants were included in this analysis (Fig 1).

### Ethics statement

The survey was approved by the Institutional Review Board of the Korean Centers for Disease Control and Prevention, and written informed consent was obtained from all participants.

### Medical history and lifestyle habits

Data on medical history and lifestyle habits were collected using self-reported questionnaires. Smoking status was categorized into 2 groups: current smokers and non- or past-smokers. Participants were asked about their average frequency and amount of alcohol consumption in the year preceding the interview. The total amount of alcohol consumed per week was obtained by multiplying the averages of the weekly frequency by the amount consumed on a single occasion. Diet in the past year was analyzed according to the intake of 3 major nutrients: fats, carbohydrates, and proteins. The total energy intake and percentage of energy from each nutrient were then calculated. Educational status was categorized into 2 groups: through high school and after high school. Regular exercise included moderate or vigorous exercise performed on a regular basis. The definition of moderate and vigorous exercise has previously been described in detail[19].

### Anthropometric measurements

The participants’ anthropometric measures were obtained by trained examiners. Details regarding the methods used to determine height, weight, waist circumference, and blood pressure are provided elsewhere[5]. BMI was calculated as the weight in kilograms divided by the height in meters squared. Muscle mass and total body fat were measured by dual-energy X-ray absorptiometry (DXA; QDR 4500A, Hologic Inc., Waltham, MA, USA). Appendicular skeletal muscle mass (ASM) was defined as the sum of the lean soft tissue masses of the arms and legs, according to the method proposed by Heymsfield et al.[21] The percentage of total body fat was calculated using the following formula: percentage of total body fat = fat mass (kg) / total mass (kg) × 100.

### Biochemical measurements

Venous blood samples were obtained after the participants had fasted for at least 8 hours. Serum samples were immediately processed, refrigerated and transported to a central laboratory (NeoDin Medical Institute, Seoul, South Korea), where they were analyzed within 24 hours. Liver enzymes, triglycerides (TG), total cholesterol, high-density lipoprotein (HDL) cholesterol, low-density lipoprotein (LDL) cholesterol, and fasting plasma glucose were analyzed using a Hitachi Automatic Analyzer 7600 (Hitachi, Tokyo, Japan). Insulin was assessed using a 1470 Wizard gamma-counter (Perkin-Elmer, Turku, Finland). Hepatitis B surface antigen was analyzed using an electrochemiluminescence immunoassay (Modular E-170; Roche diagnostics, Mannheim, Germany).

### Definitions

Heavy alcohol consumption was defined as consuming at least 210 g/week. Elevated liver enzymes were defined as levels of alanine aminotransferase (ALT) of at least 32 U/L or levels of aspartate aminotransferase (AST) of at least 34 U/L[22]. In this study, ALD was defined as heavy alcohol consumption and elevated liver enzymes.

Sarcopenia was evaluated by the skeletal muscle index (SMI), which is calculated as the appendicular muscle mass divided by height squared, i.e., (ASM)/Ht^2^. Sarcopenia was defined as an SMI more than 1 standard deviation (SD) below the sex-specific normal mean of the younger reference group[23]. The cut-off value for sarcopenia in this study, based on the value 1 SD below the mean of young adults, was 6.96 kg/m^2^.

Metabolic syndrome was defined using the criteria of the American Heart Association and the National Heart, Lung, and Blood Institute, and the cut-off point for waist circumference (greater than or equal to 90 cm for men and 80 cm for women) was determined based on the International Diabetes Federation criteria for the Asian population (2009)[24].

Insulin resistance was estimated using the homeostasis model assessment of insulin resistance (HOMA-IR)[25], with the following formula: HOMA-IR = fasting glucose (mg/dL) × fasting insulin (μU/mL) / 405.

### Statistical analysis

The data are expressed as the mean±SD or the percentage. Participant characteristics were compared using independent sample Student’s *t*-tests for continuous variables and Chi-square tests for categorical variables. The interaction between alcoholic liver disease and heavy alcohol drinking was assessed using a two-way ANOVA. The subjects were divided into 4 groups according to the presence of ALD and heavy alcohol consumption. To compare the SMI values, an ANCOVA was performed after adjusting for age, energy intake, smoking, exercise, weight, waist circumference, protein intake, and metabolic syndrome. The odds ratios and 95% confidence intervals (CIs) for sarcopenia were also calculated by multiple logistic regression analysis after adjusting for age, energy intake, protein intake, waist circumference, smoking, exercise and metabolic syndrome. We considered a *P* value < 0.05 to indicate statistical significance. Statistical analyses were performed using the survey procedure of SAS software (version 9.2; SAS Institute, Cary, NC, USA).

## Results

### Baseline characteristics

The baseline characteristics of the study participants are presented in Table 1. Of the 1,151 elderly male subjects, 1,015 were non-heavy drinkers and 136 were heavy drinkers. The mean age of the study participants was 71.6 ± 0.2 years. There were no differences between the non-heavy and heavy alcohol consumer groups in height, weight, BMI, prevalence of sarcopenia or metabolic syndrome, serum glucose, total cholesterol, triglycerides, HOMA-IR, proportion of current smokers, or educational attainment. However, compared with the non-heavy drinkers, the heavy drinkers were younger and had a higher systolic and diastolic BP and total energy intake. Heavy drinkers also had both higher HDL cholesterol and AST levels and lower LDL cholesterol. Although not significant, the group of heavy drinkers tended to have a higher waist circumference (*P* = 0.061) and to exercise more regularly (*P* = 0.060). There was no difference in SMI between the non-heavy and heavy drinkers (7.1 ± 0.0 kg/m^2^ vs. 7.3 ± 0.1 kg/m^2^, *P* = 0.145).

**Table 1:** Baseline characteristics of elderly male subjects with and without heavy alcohol consumption.

Variables	Total	Non-heavy drinker	Heavy drinker	*P* value
	(N = 1151)	(N = 1015)	(N = 136)	
Age (years)	71.6±0.2	71.7±0.2	70.6±0.5	0.025
Height (cm)	165.0±0.2	164.7±0.2	165.7±0.8	0.286
Weight (kg)	62.9±0.4	62.7±0.4	64.1±1.2	0.255
BMI^#^ (kg/m^2^)	23.1±0.1	23±0.1	23.4±0.4	0.465
Waist circumference (cm)	84.4±0.3	84.2±0.4	86±1.0	0.061
ASM (kg)	19.5±0.1	19.4±0.1	20±0.3	0.095
SMI^$^ (kg/m^2^)	7.1±0.0	7.1±0.0	7.3±0.1	0.145
Sarcopenia (%)	39.7 (1.9)	40 (2.0)	38.0 (5.0)	0.706
Total body fat (%)	22.7±0.2	22.5±0.3	22.1±0.7	0.601
Systolic BP (mmHg)	128.0±0.7	127.5±0.7	131.3±1.8	0.042
Diastolic BP (mmHg)	74.7±0.4	74.3±0.4	77.6±0.9	<0.001
Glucose (mg/dL)	105.2±0.8	104.6±0.9	109.8±2.8	0.081
Total cholesterol (mg/dL)	181.0±1.2	181.8±1.3	175.5±3.7	0.109
HDL cholesterol (mg/dL)	48.3±0.4	47.6±0.4	53±1.4	<0.001
LDL cholesterol (mg/dL)	104.9±1.2	106.6±1.2	92.1±3.9	<0.001
Triglyceride* (mg/dL)	123.2 (118.2–128.4)	121.6 (116.6–126.9)	135 (118.4–154)	0.135
ALT* (IU/L)	18.9 (18.4–19.5)	18.8 (18.2–19.4)	19.7(18.1–21.4)	0.351
AST* (IU/L)	23.6 (23.0–24.2)	23 (22.4–23.6)	28.2 (25.8–30.8)	<0.001
HOMA-IR*	2.2 (2.1–2.3)	2.2 (2.1–2.3)	2.1 (1.9–2.3)	0.279
Dietary intake				
Total energy (kcal/day)	1928.6±31.8	1889.5±34.6	2230.1±79.2	<0.001
Protein (% of energy)	13.7±0.1	13.7±0.1	14.1±0.4	0.227
Carbohydrate (% of energy)	73.5±0.4	73.6±0.4	72.8±1.1	0.444
Fat (% of energy)	12.7±0.3	12.7±0.3	13.1±0.9	0.674
Metabolic syndrome (%)	37.0 (1.8)	36.8 (1.9)	38.2 (5.2)	0.801
Current smoker (%)	25.7 (1.5)	24.7 (1.6)	32.8 (5.2)	0.116
Regular exercise (%)	23.4 (1.7)	22.3 (1.8)	31.1 (4.8)	0.060
Education (%)	30.9 (1.7)	31.6 (1.8)	25.7 (5.1)	0.297

Data are presented as the mean±SE or as the % (SE).

*Data are presented as geometric means (95% confidence interval).

^#^BMI is calculated as the weight in kilograms divided by the height in meters squared.

^$^SMI is calculated by appendicular muscle mass divided by height squared.

Abbreviations: BMI, body mass index; ASM, appendicular skeletal muscle; SMI, skeletal muscle index; BP, blood pressure; HDL, high-density lipoprotein; LDL, low-density lipoprotein; AST, aspartate aminotransferase; ALT, alanine aminotransferase; HOMA-IR, homeostasis model assessment-insulin resistance

### Adjusted skeletal muscle index in participants stratified by the presence of liver disease and heavy alcohol consumption

The participants were stratified according to the presence of liver disease and heavy alcohol consumption into the following groups: non-heavy drinkers without liver disease (group 1), heavy drinkers without liver disease (group 2), non-heavy drinkers with liver disease (group 3), and heavy drinkers with liver disease (group 4). To adjust for confounding covariates that could affect sarcopenia, we generated six multivariate logistic regression models (Table 2). After adjusting for age, total energy intake, and percentage of protein intake from nutrients (model 1), group 4 (ALD group) had a significantly lower SMI than the other groups (*P* = 0.023). In addition, when we further adjusted for other variables (model 2: model 1 plus smoking status and exercise; model 3: model 2 plus weight; model 4: model 2 plus waist circumference; model 5: model 2 plus total body fat percentage; and model 6: model 2 plus percentage with metabolic syndrome), the low SMI of group 4 remained significant in all models (all *P* < 0.05).

**Table 2:** Adjusted skeletal muscle index (SMI) according to the presence of liver disease and heavy alcohol consumption.

	Liver disease (-)		Liver disease (+)		*P* for trend
	Non-heavy drinker	Heavy drinker	Non-heavy drinker	Heavy drinker	
Model 1	7.14 ± 0.03	7.29 ± 0.11	7.14 ± 0.08	6.84 ± 0.11	0.023
Model 2	7.14 ± 0.03	7.29 ± 0.10	7.16 ± 0.08	6.86 ± 0.11	0.030
Model 3	7.15 ± 0.03	7.21 ± 0.09	7.16 ± 0.05	6.91 ± 0.07	0.002
Model 4	7.17 ± 0.03	7.19 ± 0.09	7.13 ± 0.06	6.85 ± 0.07	0.001
Model 5	7.14 ± 0.03	7.29 ± 0.10	7.16 ± 0.08	6.85 ± 0.10	0.015
Model 6	7.16 ± 0.03	7.29 ± 0.11	7.11 ± 0.08	6.83 ± 0.10	0.013

Model 1 is adjusted for age, total energy intake, and percentage of protein intake.

Model 2 is adjusted for Model 1 + smoking status and exercise.

Model 3 is adjusted for Model 2 + weight.

Model 4 is adjusted for Model 2 + waist circumference.

Model 5 is adjusted for Model 2 + total body fat percentage.

Model 6 is adjusted for Model 2 + metabolic syndrome.

**Fig 2:**
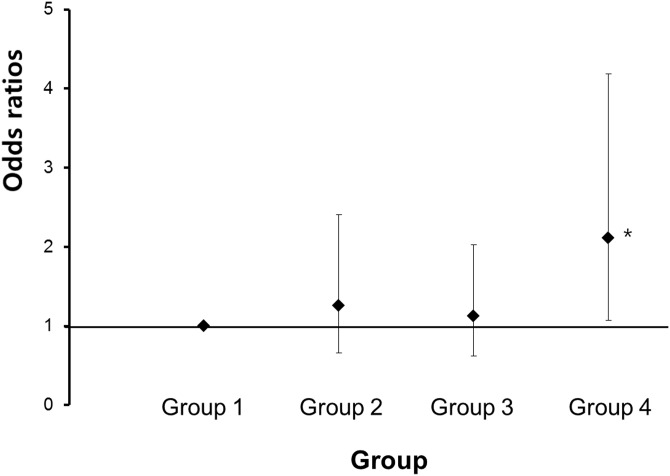
Odds ratios (95% CI) of sarcopenia according to heavy alcohol consumption and alcoholic liver disease in elderly Korean men. The odds ratio of group 4 (subjects with both heavy alcohol consumption and ALD) was significantly higher than that of group 1 (subjects without heavy alcohol consumption and ALD). Abbreviations: ALD, alcoholic liver disease.

After adjusting for age, total energy intake, protein intake, smoking, regular exercise, and waist circumference, the odds ratio for sarcopenia was 1.259 (95% CI = 0.658–2.410) in group 2, 1.121 (95% CI = 0.62–2.026) in group 3, and 2.115 (95% CI = 1.068–4.189) in group 4, compared with group 1 (Fig 2). These findings suggest that heavy alcohol consumption was a significant risk factor in the ALD group.

**Fig 3:**
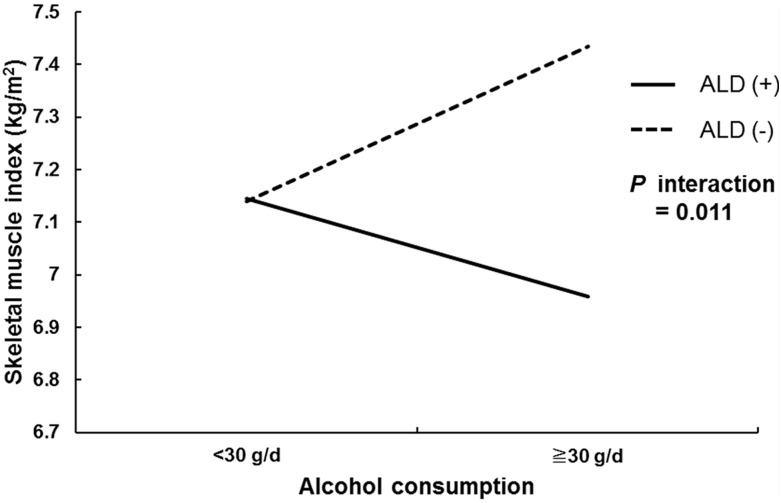
Mean SMI for subjects with and without heavy alcohol consumption. Heavy alcohol consumption led to a decrease in the SMI of subjects with ALD, while it led to an increase in SMI for subjects without ALD. The *P* value of the two-way ANOVA indicated a significant interaction. Abbreviations: ALD, alcoholic liver disease.

To test for the interaction between liver disease and heavy alcohol consumption, a two-way ANOVA was performed (Fig 3). A significant interaction was detected between liver disease and heavy alcohol consumption (*P* interaction = 0.011).

### Comparison of heavy drinkers and non-heavy drinkers according to the presence of liver disease

Because the SMI was significantly lower in heavy alcohol consumers with liver disease and changed with the interaction between liver disease and heavy alcohol consumption, we compared the characteristics between non-heavy drinkers and heavy drinkers according to the presence of liver disease (Table 3). Among subjects who did not have liver disease, the heavy alcohol consumers were younger, exercised more regularly, had a lower total cholesterol and LDL cholesterol, and had a higher waist circumference, ASM, SMI, diastolic BP, HDL cholesterol, AST level, and total energy intake compared with non-heavy alcohol consumers. There was also no difference between the two groups in the proportion of subjects with sarcopenia. In contrast, in subjects with liver disease, only HDL cholesterol, AST level, and total energy intake were significantly higher among heavy alcohol consumers.

**Table 3:** Comparison of heavy drinkers and non-heavy drinkers according to the presence of liver disease.

	Alcoholic liver disease (-)			Alcoholic liver disease (+)		
	Non-heavy drinker	Heavy drinker	*P*-value	Non-heavy drinker	Heavy drinker	*P* value
	(N = 853)	(N = 87)		(N = 162)	(N = 49)	
Age (years)	71.9±0.2	70.3±0.6	0.007	71±0.4	71.4±0.7	0.676
Height (cm)	164.7±0.2	165.9±1.0	0.269	164.8±0.5	165.1±0.9	0.755
Weight (kg)	62.6±0.4	65.1±1.6	0.116	63.3±1.0	62.0±1.7	0.511
BMI^#^ (kg/m^2^)	23±0.1	23.6±0.5	0.224	23.3±0.3	22.8±0.7	0.505
Waist circumference (cm)	84±0.4	86.8±1.2	0.026	85.2±0.9	84.5±1.4	0.682
ASM (kg)	19.4±0.1	20.5±0.5	0.019	19.4±0.3	19±0.3	0.265
SMI^$^ (kg/m^2^)	7.1±0	7.4±0.1	0.021	7.1±0.1	7±0.1	0.154
Sarcopenia (%)	39.9 (2.1)	31.8 (6.2)	0.218	40.6 (4.9)	50.9 (8.9)	0.301
Total body fat (%)	21.1±0.1	24.4±0.2	<0.001	22.4±0.3	22.6±0.5	0.492
Systolic BP (mmHg)	127.8±0.8	131.4±2.3	0.112	126±1.9	131±2.7	0.123
Diastolic BP (mmHg)	74.2±0.4	78.1±1.2	0.002	74.7±1	76.6±1.5	0.254
Glucose (mg/dL)	104±1	106.5±2.8	0.39	108.2±2.3	117±5.5	0.144
Total cholesterol (mg/dL)	183±1.4	174.9±3.3	0.026	175.3±3.6	176.8±9.4	0.881
HDL cholesterol (mg/dL)	47.5±0.5	52.1±1.7	0.009	48.2±1.2	55±2.3	0.008
LDL cholesterol (mg/dL)	108.7±1.3	95.1±3.1	<0.001	95.3±3.9	85.1±10.2	0.349
Triglyceride* (mg/dL)	117.8 (112.4–123.5)	124.5 (106.7–145.1)	0.495	144.9 (131.7–159.3)	162.0 (132.0–198.7)	0.336
ALT* (IU/L)	16.9 (16.4–17.4)	16.0 (15.0–17.0)	0.109	34.1 (31.3–37.2)	30.1 (26.1–34.8)	0.150
AST* (IU/L)	21 (20.6–21.4)	22.4 (21.2–23.6)	0.026	37.8 (35.3–40.4)	45.4 (39.7–51.9)	0.021
HOMA-IR*	2.1 (2.1–2.2)	2.0 (1.8–2.2)	0.123	2.5 (2.3–2.7)	2.3 (2.0–2.7)	0.409
Dietary intake						
Total energy (kcal/day)	1906.8±38.5	2131.8±104.2	0.047	1796.8±59	2419.8±143.1	<0.001
Protein (% of energy)	13.6±0.2	14±0.5	0.362	14.2±0.4	14.3±0.5	0.817
Carbohydrate (% of energy)	73.8±0.4	73.1±1.4	0.627	72.6±1.1	72.1±1.5	0.802
Fat (% of energy)	12.6±0.3	12.8±1.1	0.823	13.3±0.8	13.6±1.6	0.860
Metabolic syndrome (%)	35.1 (2.1)	33.8 (5.7)	0.829	46.1 (4.6)	47.6 (9.7)	0.889
Current smoker (%)	24.1 (1.7)	31.1 (6.3)	0.248	28.1 (4.2)	36.3 (8.5)	0.354
Regular exercise (%)	23.1 (1.9)	36.3 (6.5)	0.031	18.2 (3.5)	20.3 (6.9)	0.772
Education (%)	32.7 (2.1)	22.2 (5.5)	0.104	25.6 (3.9)	33.1 (9.9)	0.447

Data are presented as the mean±SE or as the % (SE).

*Data are presented as geometric means (95% confidence interval).

^#^BMI is calculated as the weight in kilograms divided by the height in meters squared.

^$^SMI is calculated by appendicular muscle mass divided by height squared.

Abbreviations: BMI, body mass index; ASM, appendicular skeletal muscle; BP, blood pressure; HDL, high-density lipoprotein; LDL, low-density lipoprotein; AST, aspartate aminotransferase; ALT, alanine aminotransferase; HOMA-IR, homeostasis model assessment-insulin resistance

## Discussion

In this study, we compared SMI according to the presence of liver disease and heavy alcohol consumption. We found that heavy alcohol consumption was associated with sarcopenia among the elderly male population with ALD and that both liver disease and heavy alcohol consumption affected sarcopenia, with significant interactions.

Skeletal muscle wasting and weakness are often observed in individuals with chronic alcohol use disorder. Excessive and prolonged alcohol intake causes myopathic lesions characterized by selective atrophy of Type II fibers, although Type I fibers are relatively protected[15]. Vargas et al. reported that alcohol accelerates muscle loss and impairs the recovery of muscle mass[26]. Thapaliya et al. revealed that muscle autophagy increases with alcohol intake and contributes to sarcopenia[27]. Despite the number of studies available, the exact mechanisms of alcoholic muscle disease remain unclear. Various conditions, such as neuropathy, malnutrition, and hormonal alteration, are known to contribute to alcoholic muscle disease[16, 28, 29]. Alcohol ingestion has been identified as an independent risk factor for alcoholic myopathy[17, 30]. Our study showed that heavy alcohol ingestion was related to sarcopenia in elderly men who have ALD, demonstrating a significant interaction with ALD. However, heavy alcohol consumption did not significantly increase the risk of sarcopenia in the population without ALD (Fig 3). This finding conflicts with a previous study that reported that alcoholic myopathy occurs independent of liver disease[15]. Therefore, further population studies are needed to investigate the relationship between ALD and sarcopenia.

Among all participants, there was no significant difference in SMI between non-heavy drinkers and heavy drinkers (7.1 ± 0.1 kg/m^2^ vs. 7.3 ± 0.0 kg/m^2^, respectively, *P* = 0.145) (Table 1). However, among subjects without liver disease, heavy drinkers had a significantly higher SMI than non-heavy drinkers (7.4 ± 0.1 kg/m^2^ vs. 7.1 ± 0.0 kg/m^2^, respectively, *P* = 0.021), while no difference in SMI was found between the two ALD groups (7.0 ± 0.1 kg/m^2^ vs. 7.1 ± 0.1 kg/m^2^, respectively, *P* = 0.154). This difference could have been caused by different baseline characteristics. Aspects of metabolic syndrome, especially waist circumference, are known to be related to sarcopenia[31]. In this study, although heavy drinkers without liver disease (group 2) had a significantly higher waist circumference than non-heavy drinkers without liver disease (group 1) (*P* = 0.026), there was no difference between the two groups in the prevalence of metabolic syndrome (*P* = 0.829), as the participants had a favorable lipid profile. However, group 2 was younger (*P* = 0.007) and exercised more regularly than group 1 (*P* = 0.031). Aging is an important cause of sarcopenia[32]. Furthermore, a previous study reported that exercise enhances muscle protein production and improves sarcopenia and physical function[33]. Therefore, group 2 was likely to have a higher SMI than group 1 because subjects in group 2 were younger and exercised more regularly. Regarding the groups without liver disease, there were no differences in the characteristics between groups 3 and 4, and there was also no significant difference between the two groups in SMI (*P* = 0.154). However, after adjusting for confounding factors, SMI was significantly lower in group 4 than in the other three groups (Table 2). This finding indicated that heavy alcohol consumption led to sarcopenia only when accompanied by ALD, and this interaction between heavy alcohol consumption and ALD is shown in Fig 3.

Recently, two population studies on the impact of alcohol consumption on sarcopenia in older men were conducted[19, 34]. These studies reported that alcohol intake was not significantly associated with sarcopenia, which contrasted with the findings of our study. A recent meta-analysis also reported that alcohol consumption was not a risk factor for sarcopenia[35]. The major difference between our study and the previous studies is that our study analyzed the relationship according to the presence of ALD. Sarcopenia is a common complication of chronic liver disease. Sarcopenia increases the risk of nonalcoholic fatty liver disease[36] and affects quality of life, survival and the development of complications in patients with cirrhosis or hepatocellular carcinoma[11, 14, 37]. As sarcopenia is closely related to chronic liver disease, ALD can also confound the relationship between alcohol intake and sarcopenia. In previous studies, the presence of ALD was not considered, and this might have led to an insignificant association between heavy alcohol consumption and sarcopenia. On the other hand, our study showed that there was a significant interaction between heavy alcohol consumption and ALD, and according to our analysis stratified by the presence of ALD, heavy alcohol consumption was significantly associated with sarcopenia among those with ALD. Therefore, heavy alcohol consumption was found to be a significant risk factor for sarcopenia in those who have ALD.

The significantly increased risk of sarcopenia that was found only in subjects with ALD may be associated with the pathogenesis of ALD and sarcopenia. Multiple pro-inflammatory cytokines, such as interleukin (IL)-6, IL-10, and tumor necrosis factor (TNF)–α, are involved in the pathogenesis of ALD[38]. Increases in nuclear factors, such as kappaB (NF-κB) and TNF-α, have been observed in ALD[39, 40]. Sarcopenia is also associated with increased pro-inflammatory cytokines and NF-κB activation[41, 42]. Hyperammonemia, which occurs from impaired hepatic ureagenesis, activates myostatin in chronic liver disease via an NF-κB-mediated mechanism[4]. Myostatin inhibits protein synthesis and increases autophagy. Additionally, acetaldehyde impairs hepatic ureagenesis[43], which induces hyperammonemia. Ethanol and its metabolites including acetaldehyde inhibit skeletal muscle protein synthesis[6] and increase autophagy[27]. Because ALD and sarcopenia have a common pathogenesis and both hyperammonemia and ethanol contribute to muscle loss, ALD may lead to a more severe progression of sarcopenia in elderly populations. However, further studies are needed to support this hypothesis.

The major strength of our study is that we used data from a large representative sample of the general elderly population in Korea. This is the first study to evaluate the associations between sarcopenia and heavy alcohol consumption according to the presence of ALD with nationally representative data. In addition, the KNHANES survey used central laboratory data and a standardized questionnaire administered by a trained examiner.

However, several limitations of our study should be recognized. First, the cross-sectional nature of this study precluded our ability to identify any cause-effect relationships between heavy alcohol consumption and ALD. Furthermore, there was a risk of recall bias, as this study used self-reported data. Second, ALD was defined only by alcohol consumption history and elevated liver enzymes. AST and ALT elevations can also be caused by non-alcoholic fatty liver disease or drugs. In addition, hepatitis C virus antibody tests were not included in the KNHANES protocol. Therefore, the groups classified as ALD might have included subjects with elevated liver enzymes due to non-alcoholic causes. However, we tried to exclude subjects with medical conditions that could influence transaminase levels and to adjust for metabolic syndrome associated with non-alcoholic fatty liver disease. Third, the clinically relevant threshold of muscle mass loss is still unknown because of its associated higher risk of disability, morbidity and mortality. Universally accepted definitions of sarcopenia have not been established, and the cut-off values vary between studies. The European Working Group on Sarcopenia in Older People (EWGSOP) reached a consensus on a clinical definition and diagnostic criteria; in it, they defined sarcopenia as the presence of both low muscle mass and low muscle function (strength or performance)[12]. However, we could not use this definition because of the lack of data on muscle function. Fourth, the criteria used for elevated AST and ALT were arbitrary. The upper limit of normal AST and ALT levels has been considered to be 40 IU/L. However, several studies have re-evaluated the upper limit of normal for aminotransferase in healthy adults. Fifth, there was no information about the duration of heavy alcohol consumption because the KNHANES survey was cross-sectional and asked about the participants’ average frequency and amount of alcohol consumption in the year preceding the interview. A longer duration of heavy alcohol consumption would contribute to more muscle mass loss. Sixth, we also lacked information on the conditions influencing muscle mass, such as diuretics, corticosteroids, and immobilization. However, in this study, we excluded subjects with chronic diseases such as thyroid disease, rheumatic disease, chronic kidney disease, and malignancy, and this exclusion should have reduced this bias. Seventh, heavy drinkers were younger than non-heavy drinkers. Age is one of the most important factors affecting sarcopenia. The younger age of heavy alcohol consumers might have been due to the early mortality of alcohol consumers, as those with early deaths could not be included in the survey. However, we tried to adjust for the effects of age in the multivariate analysis.

In conclusion, this nationwide survey of a representative sample of the Korean population showed that sarcopenia was accelerated in elderly males with ALD, with a significant interaction between alcohol consumption and liver disease. Additionally, further studies are needed to identify the underlying mechanisms of the relationship between ALD and sarcopenia.
